# Implementation of Distracted Estimation System based on Sensor Fusion through Correlation Analysis with Concentration

**DOI:** 10.3390/s19092053

**Published:** 2019-05-02

**Authors:** Ji-Yun Seo, Yun-Hong Noh, Do-Un Jeong

**Affiliations:** 1 Department of Computer Engineering Graduate School, Dongseo University, 47 Jurye-ro, Sasang-gu, Busan 47011, Korea; 92sjy02@gmail.com; 2 Department of Computer Engineering, Busan Digital University, 57Jurye-ro, Sasang-gu, Busan 47011, Korea; yhnoh@bdu.ac.kr; 3 Division of Computer Engineering, Dongseo University, 47 Jurye-ro Sasang-gu, Busan 47011, Korea

**Keywords:** Musculoskeletal diseases, Posture detection, Distracted-limit estimation (DLE Index)

## Abstract

Sitting for an extended time may cause a serious chronic disease such as a musculoskeletal disorder, or a cardiovascular disease, diabetes, or obesity. Because a consistently improper posture from early childhood to adolescence can have a number of undesirable effects on the formation of the musculoskeletal structure, learning to maintain a correct posture should be emphasized. A consistently improper posture can not only cause physical problems, it may also lead to emotional issues such as distractions, an attention deficit, and hyperactivity, and the possibility of a low efficiency and performance on assignments is high when the students have a low concentration. The present study implemented a distracted estimation system based on sensor fusion through correlation analysis with concentration that could estimate the level of distraction and prevent musculoskeletal diseases caused by poor sitting posture habits in daily life. The implemented system was designed in the form of a sitting cushion to reflect the ethological movements and characteristics of a sitting position that modern people spend a large amount of time in, and can be easily applied to existing chairs. Both algorithms installed in the system detected the center of gravity of the seated person and displayed positional changes that occurred based on the intensity of the postural changes when moving; thus, simultaneous determination of posture and impulsive behavior was possible. To evaluate the system performance, a posture determination evaluation was conducted, along with distraction estimation according to the rate of changes in posture that occur in everyday life. In addition, to evaluate performance in daily life, a movie-watching scenario was set up, and the distracted-limit estimation and concentration indices according to the rate of changes in posture were comparatively evaluated by reviewing a video of the subjects. The results of the posture determination performance evaluation through 100 posture repetitions on 10 subjects showed a high detection performance of 99.04%. The Pearson’s correlation coefficient results showed a high correlation coefficient (inverse) of r = −0.975076 and a P-VALUE =$\;1.654\times 10^{-6}$. This experiment objectively confirmed the correlation between the DLE Index (based on postural change) and the CI Index (based on EEG).

## 1. Introduction

Along with the recent development of the IT industry, most modern people are spending large amounts of their daily lives in a sitting posture in a chair or sofa during office work, meals, driving, or studying [1]. Sitting for an extended time may cause a serious chronic disease such as a musculoskeletal disorder, or a cardiovascular disease, diabetes, or obesity, because doing so strains the lower back three- and seven-times more than standing and lying, respectively. According to the WHO, 70% of global deaths are caused by chronic diseases, which are continuously increasing every year [2]. In particular, because a consistently improper posture from early childhood to adolescence can have a number of undesirable effects on the formation of musculoskeletal structure, learning to maintain a correct posture should be emphasized [3,4]. A consistently improper posture can not only cause physical problems, it may also lead to emotional issues such as distractions, an attention deficit, and hyperactivity, and the possibility of a low efficiency and performance on assignments is high when the students have a low concentration. It is therefore essential to maintain a correct posture through an improvement of sitting habits; however, self-awareness or being made aware of posture by others is very difficult in everyday life. To this end, studies [5,6,7,8] on posture analysis and correction using sensors in various forms such as chairs [9], beds [10], and shoes [11] have been conducted. In particular, chairs are one of the most useful instruments for modern people who spend most of their time sitting, although a chair-type correction system with a posture-correction function is inappropriate for use in everyday life due to problems of portability, additional costs, and the requirement of a high-performance system.

The present study implemented a distract estimation system based on sensor fusion through correlation analysis with concentration that can estimate the level of distraction to prevent musculoskeletal diseases caused by poor sitting posture habits in daily life. Most postural calibration systems are very expensive, and do not provide additional functions. The implemented system was designed in the form of a sitting cushion, to reflect the ethological movements and characteristics of a position that modern people spend a large amount of time sitting in, which can be easily applied to existing chairs. Pressure sensors were arranged in an array to reflect pressure distribution according to the sitting posture of the user, and the seat was divided into left, right, and back areas, depending on the pressure distribution. The posture of a seated person could be determined based on the pressure information of the divided areas via a triangle centroid detection algorithm and a posture determination algorithm. Both algorithms were installed in the system, and detected the center of gravity of the seated person, displaying positional changes that occurred based on the intensity of movement, and thus the simultaneous determination of posture determination and impulsive behavior are possible. The posture information allows the results of the user’s posture determination and distracted-limit estimation index to be monitored on a smartphone application via Bluetooth communication. In the present study, a posture determination experiment was conducted to evaluate the system performance. In addition, to evaluate the performance in daily life, a movie-watching scenario was set up, and the distracted-limit estimation and concentration indices, according to the rate of changes in posture, were comparatively evaluated by reviewing a video of the subject.

## 2. Materials and Methods

### 2.1. Sensor-Fusion-Based Posture Judgment System

**Figure 1:**
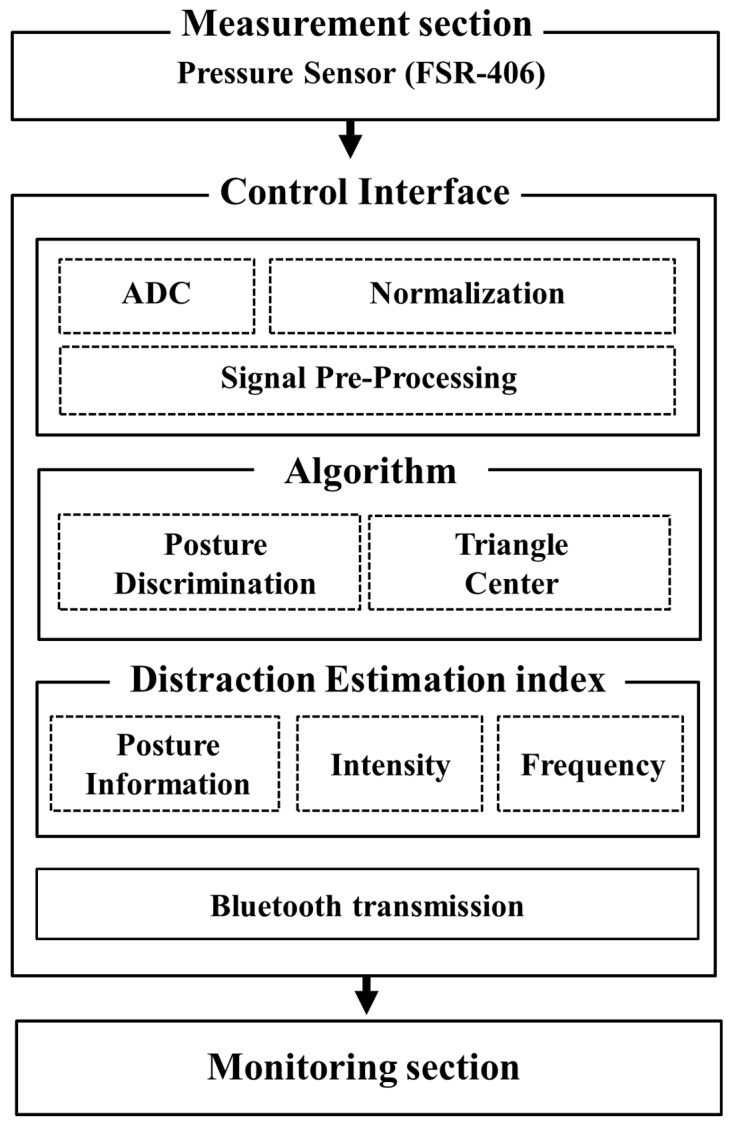
Block diagram of the overall system.

The present study implemented a posture judgment system using a sitting cushion through which the unrestrained measurement of a sitting position could be made to estimate the level of distraction in everyday life. The implemented system used pressure sensors (FSR-406, Interlink Electronics Co., California, USA) to measure the posture of the seated person. The posture determination and triangle centroid algorithms were implemented based on the pressure distribution of the sitting posture, and the distracted-limit estimation index was calculated using the rate of changes in posture of the seated person. The real-time monitoring, continuous management, and feedback of the posture information determined by the algorithms were made possible through Bluetooth communication. In addition, a real-time distraction estimation was possible through an ethological-characteristics-based distracted-limit estimation index. A block diagram of the overall system implementation is presented in Figure 1. The animal protocols used in this research were evaluated and approved by the Animal Use and Ethic Committee (CEUA) of the Institute Pasteur Montevideo (Protocol 2009_1_3284). They are in accordance with FELASA guidelines and the National law for Laboratory Animal Experimentation (Law no. 18.611).

### 2.2. Control Interface

**Figure 2:**
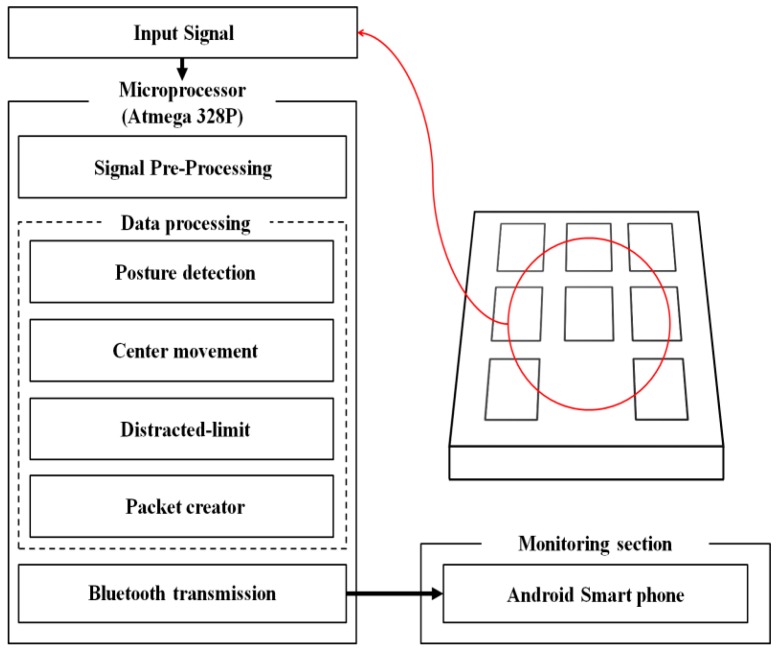
Control interface.

An Arduino Pro Mini (an ATmega 328P-based micro control board by SparkFun Electronics Co., Colorado, USA), which can produce a high performance at low voltage, was used to convert the analog pressure signal data detected from the seated posture into a digital signal. The operation of the implemented system started from the power supply, and UART was initialized for data communication. At the same time, the timer registers were initialized to set up the data sampling rate, and the analog-to-digital converter (ADC) registers that convert analog pressure signals into digital signals were applied. Once the entire controller was ready, information from each pressure sensor was transmitted through the ADC I/O port, and each bit of information was converted into a digital signal through a 10-bit ADC. The converted signals were packetized through signal processing and transmitted to the monitoring section via Bluetooth communication; thus, posture determination and measurements of posture changes were possible. Figure 2 shows the interface of the system.

### 2.3. Posture Judgment and Distraction Estimation Techniques

#### 2.3.1. Triangle Centroid Algorithm

In order to determine existing postures, a pressure-distribution-based posture determination method using a pressure sensor [12] and a skeleton data-based posture determination method using a Kinect [13] were studied. However, the techniques of previous studies do not facilitate the analysis of posture change rate and impulsive posture very well. In this paper, for an estimation of the level of distraction according to the rate of changes in posture, a triangle centroid algorithm based on the pressure distribution of the seated person was implemented. The signals used in the triangle centroid algorithm were divided into a total of three areas, namely, right, left, and back, according to the level of pressure distribution when sitting, as shown in Table 1.

The area was divided into three parts—left, right, and back, (a = left area, b = right area, c = back area)—based on the pressure distribution according to the sitting posture, and formed into an arbitrary triangle; in addition, to detect the coordinates of the center of gravity, Equation (1) was implemented. In Equation (2), the value of a movement is determined by obtaining the difference between the center of the current and previous coordinates.

(1) $$TC\left(x,y\right)=\left(\frac{-a+b+0}{3},\frac{0+0-c}{3}\right)-\left(X_{\mathrm{cp}},Y_{\mathrm{cp}}\right)$$

(2) $$MV={\mathrm{TC}}_{2}-{\mathrm{TC}}_{1}$$

**Figure 3:**
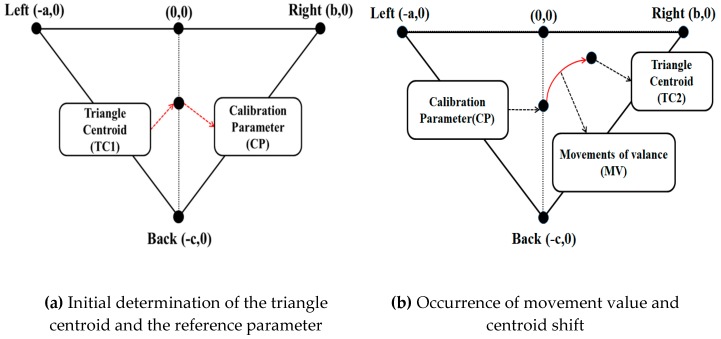
Triangle centroid detection technique.

The TC is used as the location of the center coordinates of the initial sitting posture, and initializes the coordinates produced through posture changes that occur afterward. Accordingly, the initial coordinates of Equation (1) are set to (0, 0), which are afterward set according to the postural changes. The previous TC returns the amount of changes in the x- and y-axes to the MV based on the difference from the current coordinates. Figure 3 shows the determination of the triangle centroid and the calculation technique of the movement values; the initial centroid (TC1) determined through the triangle centroid detection algorithm in (a) is represented as the reference point (CP) to determine the degree of change of the centroid (TC2), which is continuously detected afterward. Information on the shifted centroid (TC2) in relation to the initial centroid (TC1) is shown in (b). The suggested triangle centroid algorithm detects the postural changes of the seated person through the occurrence of movements and positional shifts based on the three areas divided according to the pressure distribution of the sitting posture.

#### 2.3.2. Posture Determination Algorithm

**Figure 4:**
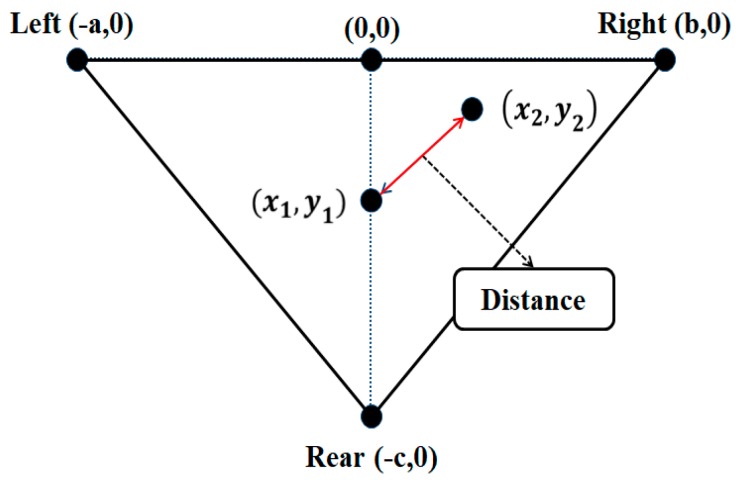
Distance calculation of postural change.

Using the triangle centroid algorithm, the detection of the centroid shift and the determination of information on the postural changes are possible; in addition, the determination of movement intensity is possible by reflecting the distance values. To measure the intensity of a movement, the distance between the current and previous centroids in the coordinates is calculated, and the distance value is reflected as the intensity of the movement. To calculate the distance between two centroids, Equation (3) is used. Figure 4 shows the distance calculation in the coordinates. To determine the posture, the current centroid was set as ($x_{1}$, $y_{1}$), the coordinates of the previous centroid were set as ($x_{2}$, $y_{2}$), and the shortest distance along a straight line between the two centroids was calculated and used.

(3) $$Distance=\;\sqrt{{\left(x_{2}-x_{1}\right)}^{2}+{\left(y_{2}-y_{1}\right)}^{2}}$$

#### 2.3.3. Distraction Estimation Algorithm based on the Rate of Postural Change

The present study classified five types of postures that are frequently used in everyday life, and determined any impulsive behaviors using the triangle centroid and posture determination algorithms. The distance between two centroids, determined by a distinguishable shift of the center of gravity using the implemented algorithms, was calculated along with the area reflecting the frequency in the time domain. Equation (4) is a distracted-limit estimation index (DLE Index) reflecting intensity, which is the gap between two centroids before and after the occurrence of a movement in the distance and time domains of the detected peaks over time. The time domain is reflected by the calculation of n areas, which are the accumulated amount of the number of occurrences and intensities, and are used as the distracted-limit estimation indices. The developed distraction index is used as the comparison target for the concentration index of the EEG, and can be used as a similar index to decrease the concentration by postural changes that occur when sitting. In addition, it has the advantage of continuous monitoring, due to its high real-time performance, and its utilization in concentration and distraction training appears to be possible through an adjustment of the time domain.

(4) $$DLE\;Index_{n}=\;\overset{n}{\underset{k=0}{\sum }}{\left(\mathrm{Distance}\times \mathrm{Time}\right)}_{k}$$

### 2.4. Estimation of the Concentration Index through EEG Measurements

In the present study, a technique was developed for determining the state of distraction of a seated person by analyzing the impulsive behaviors that occur in a sitting posture and estimating the level of distraction as reflected in the intensity and frequency of the behavior. First, a posture classification for effective measurements was conducted by analyzing the ethological characteristics that frequently occur in children with an attention deficit or attention deficit hyperactivity disorder. Afterward, a distracted-limit estimation index was created through the development and monitoring of the measurement system, and comparative evaluations using the estimated concentration index were conducted through measurements of the EEG for a performance evaluation.

**Figure 5:**
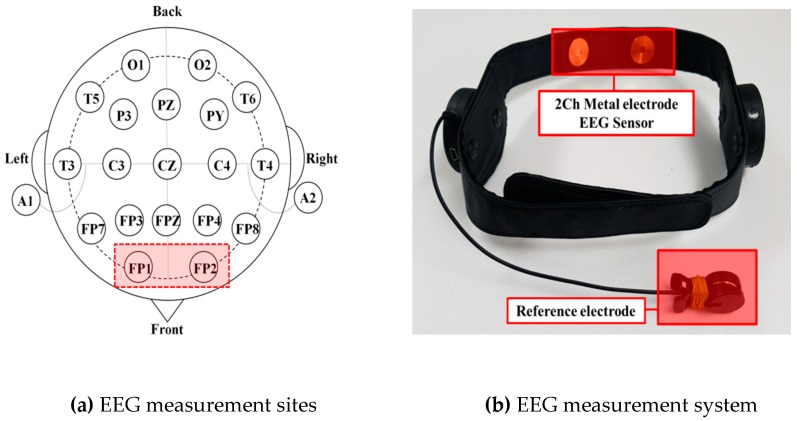
EEG measurement system and sites.

EEG can be used to quantitatively evaluate the level of concentration, and because it is used as a diagnostic tool in psychiatry, it can be used as a relative index of a distracted-limit estimation. The present study used a neuroNicle E2, a Bluetooth wireless EEG measurement instrument by Laxtha, Inc., to measure the EEG signals. This model allows two-channel raw data measurements and supports a Bluetooth-based wireless transmission. The site of the EEG measurements was the prefrontal region, and a unipolar induction method in which a ground electrode is attached to the earlobe was applied. The system used for the EEG measurements and the actual measurement sites are shown in Figure 5.

In general, the concentration was judged to be high when the SMR band and mid-β waves were high; in addition, when the θ wave—which has the characteristics of a decreasing amplitude under a distraction—was high, the level of concentration was judged to be low.

In the present study, an FFT was conducted after removing noise such as eye movement artifacts and low-frequency noise generated by body movements. Because it is difficult to analyze concentration using only FFT signals, the power spectrum was calculated to distinguish θ, the SMR, and mid-β waves for an accurate calculation of the concentration index (CI), the results of which were used to produce the CI. The produced CI digitized the ratio of waveforms that changed according to the level of concentration, and could be used to compare the distracted-limit estimation index. The equation used to obtain the concentration index is presented in Equation (5).

(5) $$CI=Power\;ratio\;of\;\frac{\mathrm{SMR}+\mathrm{mid}\beta }{\theta }$$

## 3. Results

**Figure 6:**
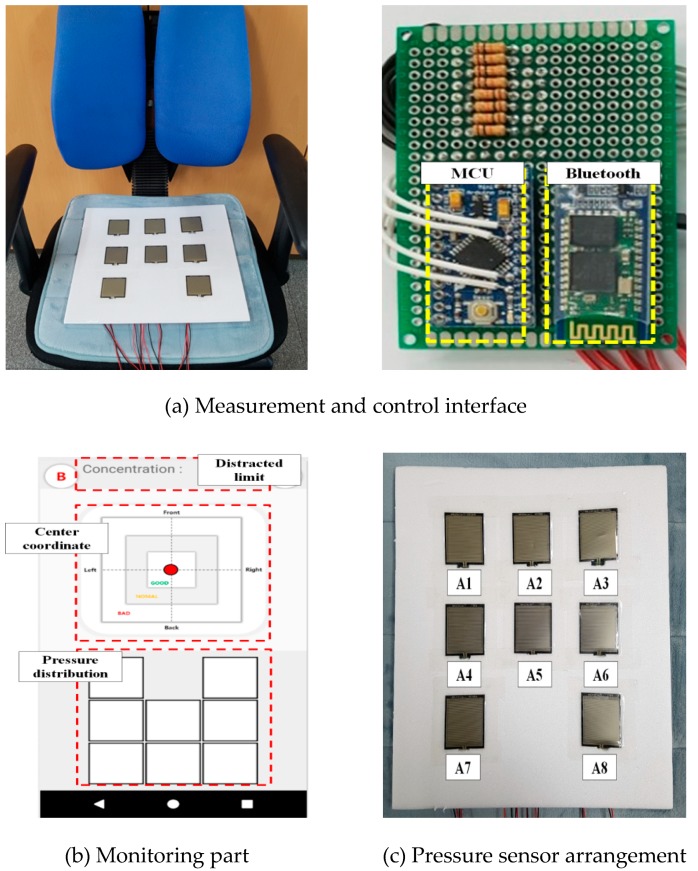
Implemented distraction estimation system.

In the present study, a distraction estimation system was implemented through posture determination and impulsive posture detection. The measurement system was designed in the form of a sitting cushion, and was divided into sitting, control, and monitoring parts. Figure 6 shows the implemented distraction estimation system, where (a) is the measurement and interface part of the system, (b) is the monitoring part of the system, and (c) is the arrangement of the pressure sensors. The implemented system can be used to easily conduct measurements in everyday life, and can be applied to an ordinary chair. In addition, posture determination and distraction estimation were made possible through unrestrained measurements when working or studying. The control interface implemented for signal processing and Bluetooth communication of the system was made up of an Arduino Pro Mini-based MCU for the signal processing and an HC-06 module for Bluetooth transmission. The monitoring part was implemented as an Android-based application to monitor the level of distraction according to the rate of postural change. With the monitoring application, the users can confirm the pressure distribution of the eight pressure sensors and coordinate information on the center of gravity based on this distribution, the rate of postural changes, the intensity of the postural changes, and the distracted-limit estimation index according to postural changes, which can be confirmed in real time.

### 3.1. Evaluation of Posture Determination Performance

**Figure 7:**
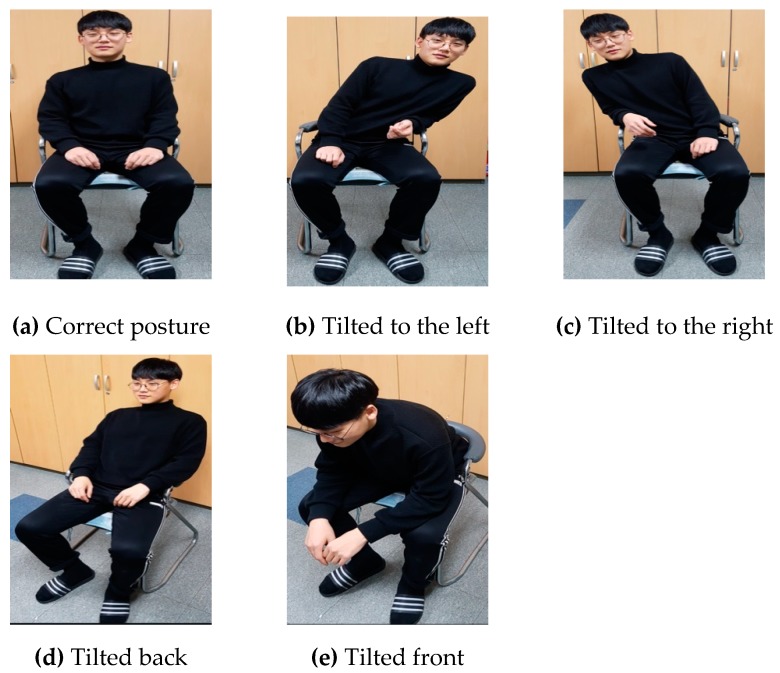
Five reference postures applied during the experiment.

For an estimation of the level of distraction, the posture determination performance of a seated person was evaluated, and the trigonometric center and posture determination algorithms were implemented to determine their posture. The reference postures for determination were five types of postures applied in everyday life, which were classified into correct posture; postures tilted to the front, back, left, and right; and right- and left-leg crossed postures. Figure 7 shows the five sitting postures used in the experiment, in which a total of 10 subjects applied each posture 100 times. The measurements of each posture are shown in Table 2. The experiment results demonstrate an overall average posture determination success rate of 99.04%, despite similarities among the right- and left-tilted and crossed-leg postures. Some of the errors appear to have been caused by limitations in the sensor recognition according to users’ body shapes and the basic error range of the sensor.

### 3.2. Performance Evaluation in Everyday Life

**Figure 8:**
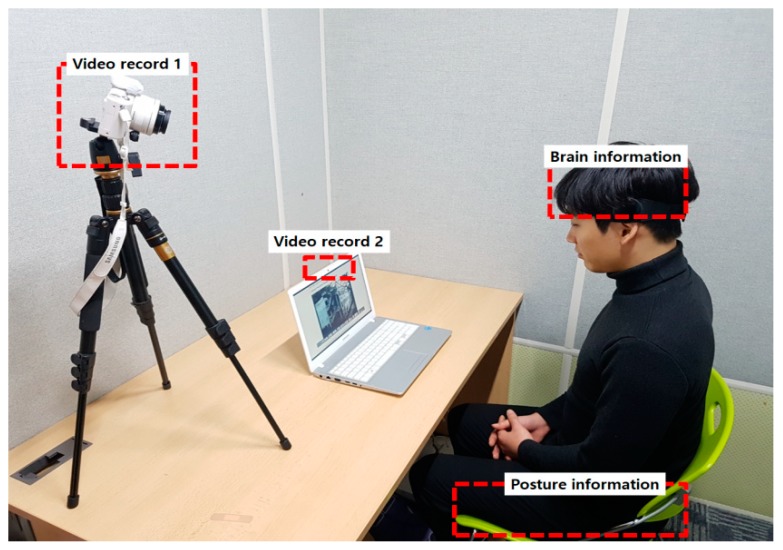
Everyday life measurements used for performance evaluation.

To evaluate the performance of the suggested distracted-limit estimation index, the rate of changes in the postures and the EEG-based concentration index were measured and compared while viewing an audio-visual material. A 100-min horror movie with a number of psychological stimuli was prepared as the audio-visual material, and the experiment was conducted by assuming an everyday life situation. In addition, video recordings were made to observe the postural changes, and used to determine the posture judgments, rate of changes in the intensity, and impulsive response at or above the baseline. Figure 8 below shows an example of the distraction estimation experiment conducted, as well as the location and status of the experiment equipment applied.

**Figure 9:**
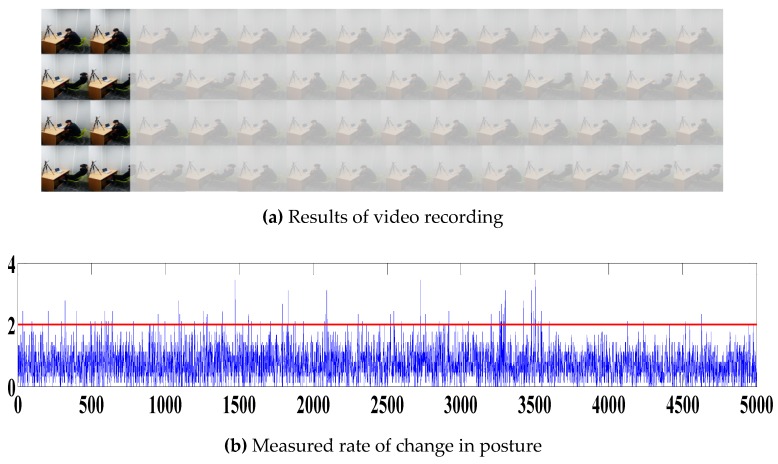

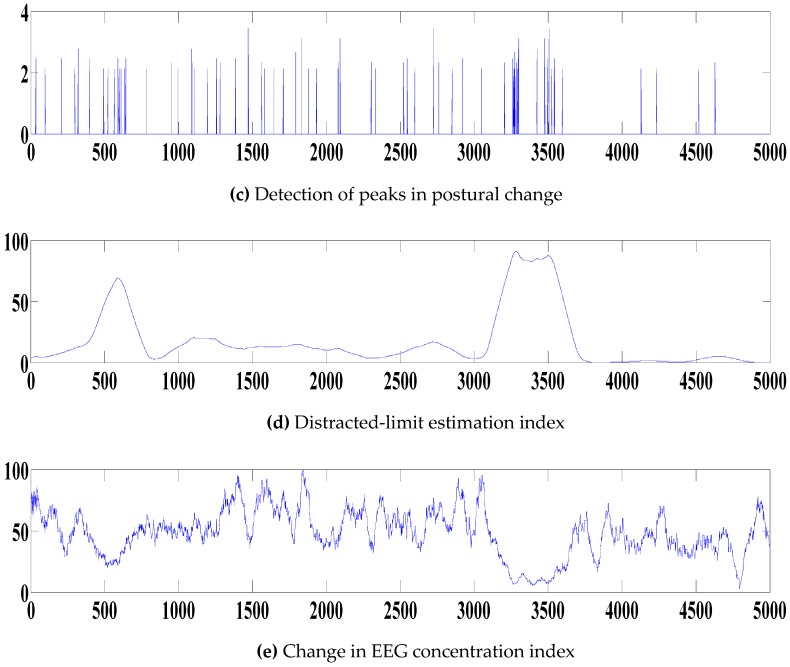
Detected amount of postural changes and peaks during the performance evaluation.

The experiment results are shown in Figure 9, in which the video recordings are presented in (a), and the intensity changes according to postural changes are shown in (b). The baseline used for a determination of the intensity of the postural change was set to 2 to control sensitivity to noise and postural changes. In (c), detected peaks of the postural change intensities above the baseline are shown, whereas changes in the distracted-limit estimation index and concentration index are presented in (d) and (e). The experiment results show that the subject demonstrated an impulsive behavior 72 times during the performance evaluation, and returned high distracted-limit estimation indices at 600 and 3000 s. When changes to the concentration index occurred where a high-level distracted-limit estimation index was detected, a relative decrease in the concentration index was determined, and a correlation between the two indices was found.

**Figure 10:**
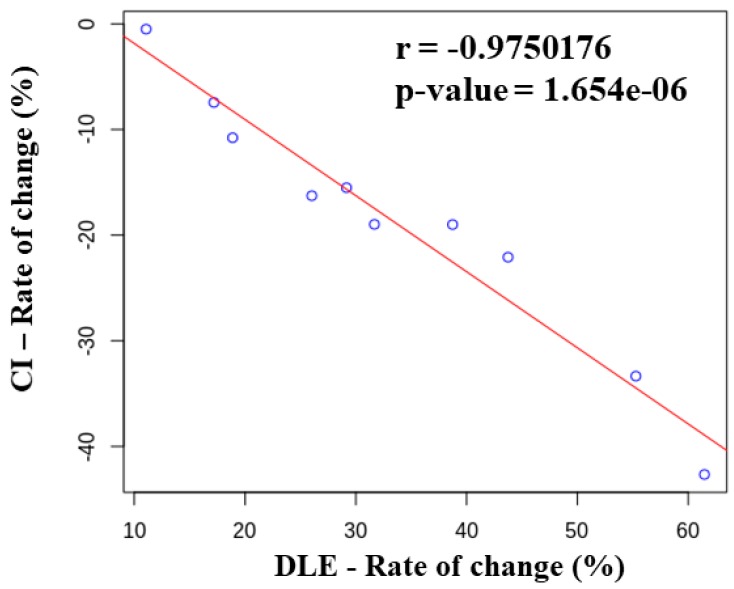
Correlation analysis between DLE Index and CI Index.

In addition, the experiment was conducted to compare the changes of the DLE Index and the CI Index according to the postural change in the same movie-watching situation with 10 subjects. The performance evaluation experiment identified the ratio of change in the DLE CI indices Before (−3 min) and After (+3 min) the section (Time) where the change in posture was most pronounced. Table 3 shows the results of the comparison of the rate of change of the DLE and CI indices. Although there was a difference in the index ratio of change between some subjects, the index ratio of change for all the subjects confirmed that the CI index decreased as the DLE Index increased. Figure 10 shows the ratio of change of the DLE and CI indices. The Pearson’s correlation coefficient results showed a high correlation coefficient (inverse) of r = −0.975076 and a P-VALUE =$\;1.654\times 10^{-6}$. This result has been verified to be statistically significant. Through this experiment, the correlation between the DLE Index (based on postural change) and the CI Index (based on EEG) was objectively confirmed.

## 4. Discussion and Conclusions

The present study implemented a distracted estimation system based on sensor fusion through correlation analysis with concentration to estimate levels of distraction and prevent musculoskeletal diseases caused by poor sitting posture habits in daily life. There have been no developed trends or research cases regarding level of distraction, and the present study attempted to reflect the disease judgment criteria for family medicine and ethological movements. The implemented system was designed in the form of a sitting cushion to reflect the ethological movements that modern people spend a large amount of time using in a sitting position. Pressure sensors were used to measure sitting posture, and the seat was divided into left, right, and back areas to determine pressure distribution during a sitting posture. The posture of a seated person was determined based on the divided areas through the application of a triangle centroid detection algorithm and a posture determination algorithm. Each algorithm detected the seated person’s center of gravity, and displayed the positional changes that occurred as the intensity of the posture changed when moving; thus, a simultaneous determination of posture and impulsive behavior was possible. Posture was determined by pressure distribution and the level of concentration, according to the distracted-limit estimation index of users, which could monitored on an Android-based smartphone through Bluetooth communication. To evaluate the system’s performance, a posture determination evaluation was conducted, along with distraction estimation according to the rate of changes in posture that occur in everyday life. The results of the posture determination performance evaluation of 100 posture repetitions by 10 subjects showed a high detection performance of 99.04%. In addition, the results of a comprehensive performance evaluation in everyday life through a video review showed a decreased concentration and increased level of distraction when a number of postural changes occurred. Although there was a difference in the index ratio of change between some subjects, the index ratio of change for all the subjects confirmed that the CI Index decreased as the DLE Index increased. The Pearson’s correlation coefficient results showed a high correlation coefficient (inverse) of r = −0.975076 and a P-VALUE =$\;1.654\times 10^{-6}$. This result has been verified to be statistically significant. Through this experiment, the correlation between the DLE Index (based on postural change) and the CI Index (based on EEG) was objectively confirmed. The implemented system not only monitors a simple sitting posture, it also estimates the level of distraction according to the rate of changes in posture, and the possibility of monitoring the level of distraction was thereby confirmed.

## Figures and Tables

**Table 1:** Area division according to pressure distribution.

Classification	Pressure sensor	Area
Left-area	A3, A5, A6, A8	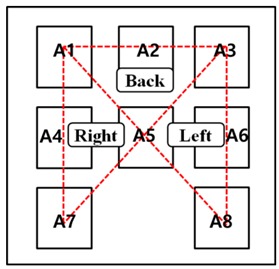
Right-area	A1, A4, A5, A7	
Back-area	A1, A2, A3, A5	

**Table 2:** Evaluation of the reference posture movements and determination performance of the system.

		**Take Posture**				
		(a)	(b)	(c)	(d)	(e)
**Detection**	(a)	100				
	(b)		100			2
	(c)			100	2.8	
	(d)				97.2	
	(e)					98
**Avg.**		100	100	100	97.2	98
		99.04				

**Table 3:** Comparison of rate of change between DLE Index and CI Index.

Subject	Time	Before		After		Rate of change (%)	
		DLE	CI	DLE	CI	DLE	CI
1	577	20.45	47.36	59.14	28.36	+38.74	−19.00
2	1113	7.27	50.58	18.35	50.08	+11.07	−0.50
3	2684	7.61	50.06	33.64	37.79	+26.03	−16.27
4	3287	20.78	56.23	82.25	13.57	+61.47	−42.66
5	1982	30.83	64.18	72.93	17.65	+55.28	−33.35
6	4832	39.46	58.44	41.33	9.65	+31.68	−18.98
7	2765	53.25	68.76	36.97	7.87	+29.16	−15.51
8	3864	7.46	57.23	26.34	46.45	+18.88	−10.78
9	2889	13.58	61.54	57.32	38.64	+43.74	−22.09
10	4312	8.94	63.72	26.13	56.28	+17.19	−7.44
**Avg.**		20.96	57.81	45.44	30.63	+33.32	−18.66
